# Ecosystem functions of fruit woody species in an urban environment

**DOI:** 10.1007/s10661-022-10717-1

**Published:** 2022-11-18

**Authors:** Fabio Orlandi, Silvia Marrapodi, Chiara Proietti, Luigia Ruga, Marco Fornaciari

**Affiliations:** Department of Civil and Environmental Engineering, University of Perugia, Borgo XX Giugno 74, 06121 Perugia, Italy

**Keywords:** Urban forest, Fruit trees, Ecosystem services, Urban green, Climate changes

## Abstract

The objective of this work was to investigate the potential ecosystem services of 16 fruit trees to plan and manage more efficiently “Urban Forest,” increasing also the resilience of cities to climate change. We evaluated the potential capacity of PM10 absorption, the storage of CO_2_ from the atmosphere, and the cooling of the environment through shading by the crown and through evapotranspiration. We observed that some species, such as *Morus nigra*, *Juglans regia*, *Pyrus communis*, and *Cydonia oblonga*, are able to store a higher quantity of CO_2_ than others over a period of 50 years, respectively, of 2.40 tons, 2.33 tons, 1.51 tons, and 0.96 tons. *Ficus carica*, *Juglans regia*, and *Morus nigra* were relevant for PM10 absorption, since they were able to absorb, referring to the year 2019, 146.4 gr/tree, 195.6 gr/tree, and 143.1 gr/tree, respectively. Results showed that these ecosystem functions depend principally on the morphological characteristics of the individuals: their height, DBH, expansion of their crowns, and characteristics of the foliage system.

## Introduction

During the last century, a rapid global urbanization has led to more than 50% of people living in cities and it is expected that by 2030, more than 60% of the total population will live in urbanized areas. The reason is that cities offer a multiplicity of services, benefits, and conveniences, but at the same time, they are the primary source of greenhouse gas emissions (https://www.epa.gov/ghgemissions/sources-greenhouse-gas-emissions) which have irreparably altered the global economic and social systems. (Clark & Nicholas, 2013). To mitigate the effects of greenhouse gases within urban areas, carbon sinks have been proposed, both inside and outside cities (Dhakal, 2010; Lazarus et al., 2013; Paloheimo & Salmi, 2013; Shigeto et al., 2012). A recent study (Park &  Schade, 2016) has shown that a fraction of CO_2_ emitted by vehicle engines was offset by photosynthesis of trees in urban green areas in Houston, Texas (Ariluoma et al., 2021).

Creating more urban green spaces that can support the wellness of the population and in the meantime meet most of their needs has become an increasingly discussed topic. Efforts are focusing especially on maximizing the contributions of a landscape to sustainable development through multifunctionality, namely, by designing areas that can simultaneously and effectively integrate multiple ecosystem services. The urban green, also intended as “Edible Forest” becomes a place for sharing, socializing, and creating a strong man-nature connection, in which the topics of myths, legends, and sayings re-emerge, a place where knowledge and memories of old traditions can be recalled, transmitted, and recovered. This wealth of knowledge has been acquired over time through direct personal experience, and it is transmitted from generation to generation mainly through oral testimonies. This knowledge represents a fundamental heritage for the survival of human societies as it is intimately associated with the perception that the people of a community have about the environment in which they live. However, this is threatened by rapid socio-economic changes and the progressive disappearance of rural societies. In this sense, ethnobotany can provide the tools for the documentation of this knowledge and therefore help understand the different systems of local knowledge, so that they can be recorded, compared, and studied. The recovery and study of this popular knowledge can also be an opportunity to develop new activities aimed at enhancing and protecting the territory (Signorini et al., 2007). The valorization and the defense of a territory look towards the reutilization of some “forgotten” tree species or minor fruit trees, which risk vanishing definitively over time. Their use is having an increasing interest from the scientific researchers because, with their genetic heritage handed down in millennia of history, these trees allow the development of strains naturally resistant to extreme climatic conditions and diseases, ensuring a fast growth with good yields without using pesticides. To strengthen the green infrastructure of cities and adapt to climate change, many countries have developed various strategies, particularly focusing on safeguarding biodiversity (Faivre et al., 2017), (https://naturvation.eu).

So, the main objective of this work was to investigate the potential ecosystem services of 16 fruit woody species to plan and manage more efficiently an “Urban Forest,” also increasing the resilience of cities to climate change. Trees are able to provide different ecosystem services such as CO_2_ storage, absorption of PM10 in the air, cooling of the area under the canopy through shading, and evapotranspiration. Atmospheric carbon is stored within trees that act as a sink, so a greater presence and quantity of trees can slow the accumulation of carbon in the atmosphere (Nowak, 1993). Urban trees have different cooling intensities, which can change among different tree species, depending on the size of the plants, and other factors such as leaf area index, leaf thickness, canopy density, light permeability, and also photosynthesis (Ballinas & Barradas, 2016; Legese Feyisa et al., 2014).

Urban air temperature can also be reduced through evapotranspiration, a process by which some of the energy absorbed by plants evaporates water inside their leaves, cooling them. Plants are capable of removing different size fractions of PM particles from the atmosphere, which are deposited on the surfaces of leaves and branches (Litschke & Kuttler, 2008); the efficiency of this process depends on the morphological and physical characteristics of the leaves themselves, which are different among different tree species (Freer-Smith et al., 2004; Mitchell et al., 2010; Mo et al., 2015; Sæbø et al., 2012; Yang et al., 2015).

Moreover, the edible fruit woody plants in the urban green areas can provide raw materials to satisfy the primary food needs; fruits have different food functions and can also have a certain nutraceutical value (presence of substances that can improve our health and delay the aging process increasing life expectancy). Edible woody fruit plants are being used as a source of food within a project, “City Fruit,” initiated in Seattle, in which urban landscapes, fruit growing, awareness of climate change, and the role of fruit trees in cities are being promoted, emphasizing their value and aiming to build larger and larger communities to actively collaborate on the project. The aim is to involve more city neighborhoods in fruit picking, encouraging proper tree management (McLain et al., 2012). City Fruit collected about 25,000 kg of unused fruit from Seattle’s urban fruit trees in 2016 and subsequently donated 13,600 kg to food banks and community organizations. The total estimated value of the harvested fruit is $60,000 (http://www.cityfruit.org).

The following work was aimed at investigating the potential ecosystem benefits, in urban environment, of 16 edible fruit woody plants utilized in a “virtual” food forest and inserted in a web application for their development evaluation. More specifically, we focused on the potential CO_2_ stock, PM10 absorption, and the cooling effect brought about by the trees’ canopies and evapotranspiration. Future investigations will evaluate the productive, economic, and ecosystem potential of fruit trees in urban environments.

## Materials and methods

### Selection of edible fruit woody plants and simulation of a new urban forest planting through a web application “Lifeclivuttreedb”

The choice of edible fruit woody plants to be planted virtually in the city was made by considering the tree diameter speed of growth, the environment in which they will grow, and particularly their ability to withstand the temperatures in Central Italy, based on their hardiness and adaptability to climate change. Sixteen species of fruit trees were identified, and their ecosystem functions assessed through the use of an online platform called “Lifeclivuttreedb.” The species are *Arbutus unedo*, *Corylus avellana*, *Cydonia oblonga*, *Diospyros kaki*, *Ficus carica*, *Juglans Regia*, *Malus Domestica*, *Malus sylvestris*, *Mespilus germanica*, *Morus nigra*, *Pyrus communis*, *Prunus avium*, *Prunus cerasifera*, *Prunus domestica*, *Prunus dulcis*, and *Sorbus domestica*.

The environment open-source software (called “Lifeclivuttreedb”) is based on web architecture and can be used on the Internet from any device, both fixed and mobile. The software “Lifeclivuttreedb” has been created within the Life Clivut project “Climate value of urban trees” which operates in four cities of the Mediterranean area: Perugia and Bologna in Italy, Thessaloniki in Greece, and Cascais in Portugal (https://www.lifeclivut.eu/). The objective of the project is the planning and management of urban green spaces, to increase resilience to climate change. To evaluate the climatic and ecosystem performance of trees in the city, we have used specific indicators: the species, the presumed age of the trees, the health status, the size of the crown, the CO_2_ storage, the capacity to reduce PM10 in the air, the cooling effect, and the biodiversity present. The values of these indicators will be calculated based on the growth of the species in future periods, through the use of specific “growth curves” that allow simulating the morphological development. In the platform “Lifeclivuttreedb” (http://lifeclivut.treedb.eu/login.php), which is accessed through a web app, it is possible to report in a geo-localized way the information on trees present in public and private relevant areas via various tools such as smartphones, tablets, and computers. This allows the creation of a database updated on the tree heritage of each city. Dendrometric data considered by the web app are the DBH (tree diameter at 1.30 m), the tree height, the height at the first stage of the branches, the size, the shape, and the transparency of the crown. The species of the tree and its phytosanitary status are also recorded, which are useful to identify possible problems for its management (Ventura et al., 2021). The “planting” of nursery individuals belonging to the fruit species was simulated in the web app.

**Fig. 1:**
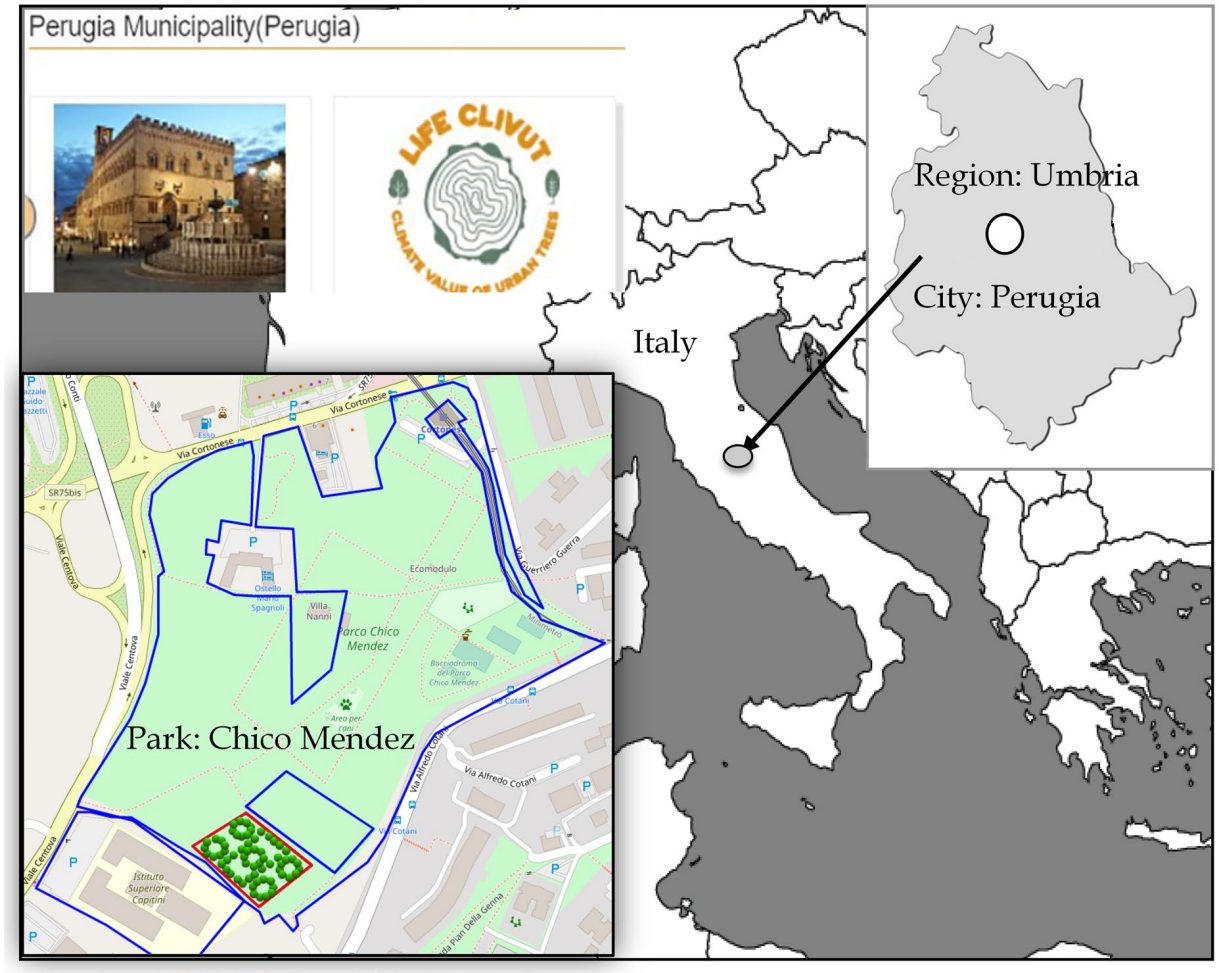
New edible fruit woody plants inside “Chico Mendez” Park, Perugia

The new virtual planting was realized in an area of Chico Mendez Park, in the city of Perugia (Fig. 1), central Italy. To realize our urban forest, 16 young species of edible fruit woody plant, about 5 years of age, were planted, 4 individuals for each species, for a total of 64, at a distance of about 5 m from each other. The site of realization of the “virtual” urban forest has an area of 6,362 m^2^. The successive ecosystem service evaluations will be realized considering both young (5 year old) and adult (30 year old) edible fruit woody plants.

### Assessment of CO_2_ storage and sequestration potential

The allometric equations used to calculate CO_2_ storage have 2 essential parameters: DBH and plant height (*H*). The platform “Life Clivuttreedb” can provide an estimate of carbon stored (C storage) expressed in tons of CO_2_ equivalent based on species and DBH, estimating the height and age of each tree. Emission sequestration is estimated based on tree biomass through the volumetric equations. The volume of tree fresh biomass is converted to dry weight through the densities of the different woods, and the carbon present is estimated and converted to stored carbon dioxide equivalent (Ventura et al., 2021). The model is based on “i-Tree” experience which calculates the total carbon storage of the urban forest and individual trees from the allometric biomass equations of forest-grown trees with the adaptation to the urban reality (Nowak, 1994, 2020; Nowak & Crane, 2000). In “Lifeclivuttreedb,” the formulas were adapted to the Mediterranean area edible fruit woody plants, estimating differences in behavior between plants with growth in natural environments and those with growth rates conditioned by a limiting urban environment. The estimation of biomass and carbon stock of tree species in the Mediterranean area was based also on the results of an Italian research program (ri.selv.Italia) funded by the Department of Agriculture and Forestry of the Italian Government (Bianchi, 2004). Dry weight biomass and stored carbon are calculated incorporating subsurface biomass by multiplying epigeal biomass by 1.28 (Sinacore et al., 2017; Tritton & Hornbeck, 1982); then, biomass is converted to kilograms of carbon (C) by multiplying by the constant 0.50 (Lamlom & Savidge, 2003), while stored carbon was converted to stored carbon dioxide (CO_2_ in ton) by multiplying by the constant 3.67 (molecular weight of CO_2_). In this study, allometric equations for estimating tree volume were selected from several sources to better fit tree modeling in the different areas analyzed (McPherson et al., 2016; Zianis et al., 2005).

### PM absorption assessment

For each tree surveyed, the atmospheric data from the station closest to the tree are used, so that they are representative of the climatic and environmental conditions in the area object of study. To calculate PM10, the “UFORE” (Urban Forest Effects Model) formula is used to estimate monthly and total annual PM10 uptake (Nowak & Crane, 1988) considering a leaf area index derived from allometric equations that predicted tree and crown height, crown diameter, and leaf area using DBH (McPherson et al., 2016).

$$\mathrm{Total\ PM10\ removed}\left(\upmu \mathrm{g}/{\mathrm{cm}}^{2}\ \mathrm{for\ year}\right) = \Sigma\ \mathrm{Vd} * \mathrm{C} * 3600 * 24 * \mathrm{Ti} * \mathrm{LAI} * \mathrm{Fd}$$

The other parameters that are considered for estimating PM absorption are as follows: deposition rate (Vd), which is set differently depending on the leaf structure and depends on the leaf conformation and the presence of trichomes (Beckett et al., 2000; Freer-Smith et al., 2004), monthly PM10 concentration (μg/m^3^), acquired by the Regional Agency for Environmental Protection (ARPA-Umbria). The calculation of PM uptake was done considering the pollutant concentrations recorded in 2019 (the year the CLIVUT Project began). The number of monthly days the leaves are present is also considered (Ti, the period considered was April–October for deciduous broadleaf trees) and the coefficient of the particulate resuspension rate of 0.5 (Zinke et al., 1967). Another parameter is the leaf area index (LAI): a dimensionless quantity obtained from the estimated leaf area (through allometric equations) and the measured crown projection of each tree. Urban tree growth equations predicted tree height, crown height, crown diameter, and leaf area using DBH (McPherson et al., 2016). The last parameter is leaf density (Fd%) which estimates the actual presence of leaves. Results were expressed as annual grams of PM10 absorbed by a tree (g/tree) to make the comparison easier. The absorption potential of trees depends on several morphological and physical characteristics of the tree and on the environment considered. Pollutant flux (*F*) was calculated as the product of the deposition rate (Vd) and the pollutant concentration (*C*) (Nowak, 2020; Nowak & Crane, 2000): *F* (g/m^2^/s) = Vd * *C*.

The total amount of PM10 removed from each tree was obtained by integrating the monthly average pollutant flux over the annual series (Bottalico et al., 2016; Manes et al., 2014), with the estimated presence of tree leaves.

### Shade effect of tree crowns

In “LifeClivutTreeDB,” morpho-geographic parameters were perfected to simulate the ground projection of crowns. This will produce an estimate of the actual energy savings on cooling and heating because of tree shade in urban areas. To calculate the potential shadow effect of the tree crown, the individual volume (calculated with the shape of the crown and the dimensions of height and width) and the crown density parameters were considered. The total shaded area is estimated as the summation of all trees present without crown interference using spatial operators directly on a GIS (Geographical Information System)-based web app (Ventura et al., 2021). Clivut-Treedb used is based on the GIS platform (Esri Inc. 2020. ArcGIS Pro Version 2.5. Esri Inc. https://www.esri.com/en-us/arcgis/products/arcgis-pro/overview). For each tree, a circle is considered with the center at the position of the tree and a diameter equal to the size of the crown. This produces an image with intertwined circles, which means that it is not possible to calculate the coverage area by adding up the area of each circle since the areas that overlap between the various circles would be counted multiple times. Using a spatial operator to determine the intersections between multiple circles allows eliminating duplications, generating, as a result, a complex geometry consisting of all polygonal geometric shapes obtained by the “fusion” of all circles in which the overlaps are eliminated. In the end, we obtain a geometric multipolygonal shape in the form of a “cloud,” which represents the area covered by the crowns of the trees. The GIS that performs this operation was used to consider the projected areas of actual shade derived from trees in urban green areas.

### Cooling potential through shading (ΔT)

Lin and Lin (2010) studied the cooling capacity of urban trees commonly planted in subtropical areas, and they found that the features that made the greatest contribution to reducing below-crown air temperatures during the day were as follows (in descending order of importance): leaf color, leaf area index (LAI), leaf thickness, and leaf roughness. Leaf color and roughness (often dictated by the presence or absence of hair or by the characteristics of wax on the leaf surface) can influence leaf and air temperatures through their effect on the leaf’s ability to reflect radiation (Monteiro Vaz et al., 2016). Leaf roughness, shape, and size can affect water supply and energy transfer (Nicotra et al., 2011; Schuepp, 1993). The use of the online platform “LifeClivutTreeDB” allowed to express the cooling capacity of different edible fruit woody plants whose cultivation in urban areas is hypothesized to reduce the temperature under the crown at a height of about 1.5 m from the ground (Lin & Lin, 2010).

### Evaluation of urban tree growth curves

The quite uniform growth conditions in rural forests allow to generate growth equations specific to each tree species, which is more difficult for trees in “urban forests” because of their heterogeneity. In our experience, a series of allometric equations were used to interpret plant growth of tree species derived from the analysis of empirical observations made by the U.S. Forest Service (McPherson et al., 2016). The various allometric equations tested included logarithmic and exponential equations, as well as four polynomial models (linear, quadratic, cubic, and quartic). Parameters predicted by the models include the following: the use of tree age to predict DBH and “viceversa” and the use of DBH to predict tree height, crown height and diameter, and leaf area. To establish fixed relationships between the different parameters (AGE = Age, DBH = Diameter at man chest, Crown Diameter = Crown Diameter, Height = Height, Leaf area = Leaf area) and to eliminate abnormal growth values, starting from the selected equations, one-input “Growth Matrices” were defined to derive a variable based on a predictor. The matrices used to evaluate volume accretions can predict DBH values based on individual age (AGE predicts DBH) and height values derived from the DBH (DBH predicts HEIGHT). For the AGE and DBH relationship, a single matrix was used to predict DBH from individual age and to derive the age value based on the DBH of a given species. From the allometric growth equations, 2 matrices with one input were defined to derive “Crown diameter” and “Leaf area” from the DBH. The matrices created provided the basis for evaluations regarding the dimensional development of the crown projected to the ground and the tree volume to calculate stored carbon.

### Dynamic assessment of CO_2_ stock by edible fruit woody species

The values of the growth matrices for DBH and height for individual species were used in the allometric equations for estimating tree volumes and progressive CO_2_ sequestration. For each tree species considered, thanks to the results of the equations, we proceeded to construct a matrix that created a correlation between the variables AGE and DBH and the values of “CO_2_ storage” during the entire biological life of the individual (from the first year of planting to the 50th year). In this way, for each tree species, it is possible to obtain, according to dimensional growth models, the accumulation of CO_2_ over time and the annual sequestration, showing the maximum value of increase during the life of the edible fruit woody plant.

## Results

The morphological characteristics of the individuals of each considered species for the new planting were defined considering the dendrometric average values derived from the infield experience on plants of different development by Life Clivut census activities http://lifeclivut.treedb.eu/login.php (Table 1).

**Table 1:** Average morphological values per edible fruit woody plants

Species	Scientific name	Height (m)	Branch height (m)	DBH (cm)	Crown diameter (m)	
					Max	Min
*Arbutus unedo*	Strawberry	1.8	1.1	5.3	1.7	1.2
*Corylus avellana*	Common hazel	1.9	1.2	5.8	1.8	1.2
*Cydonia oblonga*	Quince	1.9	1	5.8	1.8	1.4
*Diospyros kaki*	Persimmon	1.9	1.1	5.7	1.7	1.1
*Ficus carica*	Common fig	1.8	1	5.9	1.6	1.1
*Juglans regia*	Walnut tree	1.7	0.8	5.3	1.6	1.2
*Malus communis*	Apple	1.9	1.1	5.6	1.7	1.2
*Malus sylvestris*	Crab apple	1.8	1.2	5.5	1.7	1.1
*Mespilus germanica*	Medlar	1.9	1	5.5	1.8	1.3
*Morus nigra*	Black	1.9	1	5.8	1.8	1.2
*Prunus avium*	Sweet cherry	2.3	1.3	5.7	1.9	1.3
*Prunus cerasifera*	Cherry plum	1.8	0.9	5.2	1.9	1.3
*Prunus domestica*	Plum	1.8	1.1	5.2	1.8	1.2
*Prunus dulcis*	Almond	1.7	0.9	5.2	1.5	1.4
*Pyrus communis*	Common pear	1.8	0.8	5.3	1.7	1.2
*Sorbus domestica*	Service tree	1.7	0.9	5.2	1.7	1.1

### CO_2_ storage and sequestration potential with dynamic assessment by edible fruit woody plants

Estimates of average CO_2_ accumulation for the species considered, 50 years from the time of urban forest establishment, are presented in Table 2. The average CO_2_ accumulation per species at the time of implantation is very low, only 0.07 tons. Over the years, the potential for trees to store CO_2_ increases as DBH and age increase. Among all the species considered, *Ficus carica* is the one that by the age of 50 comes to store the highest amount of CO_2_, namely, 2.93 tons. The average CO_2_ stock for species at 50 years is 1.32 tons while the sum for “urban forest” reaches 84.28 tons.

**Table 2:** Average CO_2_ accumulation (ton) by species at implantation (5 years) and in following years

Species	DBH	Age	CO_2_ (5 years)	CO_2_ + 10 years	CO_2_ + 20 years	CO_2_ + 30 years	CO_2_ + 40 years	CO_2_ + 50 years
*Arbutus unedo*	5.4	3.8	0.06	0.24	0.42	0.60	0.77	0.94
*Corylus avellana*	5.8	3.3	0.04	0.15	0.26	0.40	0.59	0.82
*Cydonia oblonga*	5.8	4.8	0.09	0.27	0.45	0.63	0.80	0.96
*Diospyros kaki*	5.7	4.7	0.08	0.26	0.44	0.62	0.79	0.95
*Ficus carica*	5.9	6.1	0.23	0.67	1.18	1.71	2.29	2.93
*Juglans regia*	5.3	5.4	0.07	0.23	0.57	1.09	1.69	2.33
*Malus communis*	5.6	3.9	0.05	0.16	0.27	0.41	0.60	0.84
*Malus sylvestris*	5.6	3.6	0.04	0.16	0.26	0.41	0.60	0.83
*Mespilus germanica*	5.5	3.9	0.04	0.20	0.44	0.72	1.02	1.34
*Morus nigra*	5.8	6.2	0.12	0.40	0.80	1.27	1.80	2.40
*Prunus avium*	5.8	3.4	0.05	0.23	0.42	0.59	0.76	0.93
*Prunus cerasifera*	5.3	3.1	0.05	0.23	0.42	0.59	0.76	0.93
*Prunus domestica*	5.2	3.1	0.05	0.23	0.42	0.59	0.76	0.93
*Prunus dulcis*	5.2	3.1	0.05	0.23	0.42	0.59	0.76	0.93
*Pyrus communis*	5.4	2.1	0.04	0.28	0.67	0.99	1.26	1.51
*Sorbus domestica*	5.3	2.1	0.04	0.28	0.67	0.99	1.26	1.51

Average values			0.07	0.26	0.51	0.76	1.03	1.32
Σ “food forest”			4.46	16.95	32.35	48.76	66.01	84.28

### PM uptake assessment

The average annual absorption of PM10 was evaluated (Table 3) by estimating the g/cm^2^ of the leaf, denoted as grams of PM10/tree, that trees newly planted and at the age of 30 are managing to capture. *Juglans regia* is the species that can absorb the most PM10 during both the young and adult phases; at 30 years old, it can absorb up to 195.60 g of PM10 per tree annually. The species that absorbs the lowest amount of particulate matter is *Arbutus unedo*, both young with only 6.13 g and at age 30 with the absorption of only 61.04 g. All young species absorb an average of 14.63 g of PM10 per year. The adult species instead manage to absorb an average of 110.92 g.

**Table 3:** Annual PM10 uptake (averages by species in 2019)

Species	Leaf surface (µg/cm^2^)		Tree total uptake (gr/tree)	
	Young*	Adult*	Young*	Adult*
*Arbutus unedo*	3.4	83.4	6.1	61
*Corylus avellana*	5.1	96.9	12.8	117.8
*Cydonia oblonga*	7.9	101.2	21.3	104.5
*Diospyros kaki*	5.3	89.0	18.8	98.7
*Ficus carica*	7.9	132.5	15.7	146.4
*Juglans regia*	25.5	203.2	28.6	195.6
*Malus communis*	5.6	179.8	20.3	157.5
*Malus sylvestris*	5.2	166.8	19.8	121.4
*Mespilus germanica*	2.5	78.9	9.8	82.8
*Morus nigra*	10.1	125.9	15.5	143.1
*Prunus avium*	7.3	93.4	11.2	106.7
*Prunus cerasifera*	6.6	77.9	9.6	88.4
*Prunus domestica*	3.7	67.9	9.4	71.1
*Prunus dulcis*	4.2	62.4	10.0	66.8
*Pyrus communis*	7.3	76.1	10.4	80.2
*Sorbus domestica*	9.3	116.1	14.9	132.7

Average values	7.3	109.5	14.6	110.9
Σ “food forest”	468.1	7005.6	936.5	7098.7

^*^Young individuals (5 year old); adult individuals (30 year old)

### Cooling potential through shading (ΔT)

The cooling potential was calculated by knowing the shading created by the projection of the tree crown onto the ground (Table 4). Based on the results of CLIVUT’s platform models, about half of the urban forest will be shaded when the individuals are “adult” (3405.2/6362 = 54%). Young individuals manage to shade with their canopies an average of 3.5 m^2^ each inside the considered area. Young individuals of *Juglans regia* shade only 5.3 m^2^, while as adults, the projection of their foliage on the ground reaches 107.4 m^2^ and represents the species with the highest potential for cooling through shading.

**Table 4:** Edible fruit woody plant shading effect

Species	Crown projection (m^2^)	
	Young*	Adult*
*Arbutus unedo*	2.2	16.3
*Corylus avellana*	2.3	39.4
*Cydonia oblonga*	3.1	28.5
*Diospyros kaki*	2.4	27.2
*Ficus carica*	5.8	76.6
*Juglans regia*	5.3	107.4
*Malus communis*	3.7	38.1
*Malus sylvestris*	3.4	32.5
*Mespilus germanica*	3.6	65.8
*Morus nigra*	4.4	72.5
*Prunus avium*	3.8	93.4
*Prunus cerasifera*	3.6	70.8
*Prunus domestica*	3.2	51.9
*Prunus dulcis*	2.8	34.9
*Pyrus communis*	2.7	41.5
*Sorbus domestica*	3.7	54.5

Average values	3.5	53.2
Total “Forest” (× 64)	224	3405.2

^*^Young individuals (5-year old); adult individuals (30-year old)

### Cooling potential through evapotranspiration

Through the online platform, it was possible to obtain data on the cooling potential through evapotranspiration of the 16 fruit tree species considered (Table 5) and to estimate the daily Watts of energy that both young and adult edible fruit woody plants can absorb. The potential for heat reduction was evaluated through the quantity of heat needed to evaporate the water, and it is expressed in Watts per tree. The average cooling in Watts through evapotranspiration across the “Site” for young species is only 38.89 W per day compared to an average per individuals of 1,460.91 W per day for adult species.

**Table 5:** Average daily value of Watt absorbed (cooling effect) for the different species

Species	ETP (Watt/day)	
	Young*	Adult*
*Arbutus unedo*	33.7	1128.9
*Corylus avellana*	37.1	1379.1
*Cydonia oblonga*	45.7	1308.3
*Diospyros kaki*	35.1	1488.6
*Ficus carica*	34.8	1273.7
*Juglans regia*	36.6	4135.1
*Malus communis*	38.7	629
*Malus sylvestris*	33.5	703.6
*Mespilus germanica*	42.1	2987.6
*Morus nigra*	39.2	2203.6
*Prunus avium*	47.0	661.6
*Prunus cerasifera*	47.6	837.4
*Prunus domestica*	37.7	738.2
*Prunus dulcis*	37.4	782.4
*Pyrus communis*	41.3	1343.5
*Sorbus domestica*	35.1	1774

Average value	38.9	1461

^*^Young individuals (5 year old); adult individuals (30 year old)

### Cooling potential by the temperature gradient (ΔT)

The average Δ*T* for all the species considered was calculated for young species (letter A) and adult (letter B) during the summer period from May to September (Table 6). The temperature differential between the area under the canopy and the uncovered area was evaluated considering the area covered by the crown of the 16 species of the study, each represented by 4 individuals (64 in total) and comparing it to the total cover of the urban forest on a total area of 6,362 m^2^. Adding up the percentage cover of all the species evaluated (considering 4 individuals/species), we reach a cover of 3.52% with young edible fruit woody plants and 53.52% with adult individuals. For each species (represented by 4 individuals), the *T* under the shaded area was evaluated, summing the data of all species obtaining a total value of temperature reduction in the forest of 0.06 °C with young species and 1.16 °C with adult species.

**Table 6:** Average Δ temperature for all the species during summer (from May to Sept.). Crown surface of different species and their percentages vs total forest area (6362 m^2^). Δ temp. of each species/area

Species		Medium Δ temp. (°C) May–Sept	Tot. crown surface *	% species area/forest	Δ temp. (°C) in the forest
*Arbutus unedo*	A	1.26	8.8	0.14%	0.002
	B	2.35	65.2	1.02%	0.024
*Corylus avellana*	A	1.22	9.2	0.14%	0.002
	B	1.91	157.6	2.48%	0.047
*Cydonia oblonga*	A	2.17	12.4	0.19%	0.004
	B	3.22	114	1.79%	0.058
*Diospyros kaki*	A	1.95	9.6	0.15%	0.003
	B	2.36	108.8	1.71%	0.040
*Ficus carica*	A	1.71	23.2	0.36%	0.006
	B	2.25	306.4	4.82%	0.108
*Juglans regia*	A	3.25	21.2	0.33%	0.011
	B	1.39	429.6	6.75%	0.094
*Malus communis*	A	1.93	14.8	0.23%	0.004
	B	3.13	152.4	2.40%	0.075
*Malus sylvestris*	A	1.96	13.6	0.21%	0.004
	B	2.62	130	2.04%	0.053
*Mespilus germanica*	A	1.21	14.4	0.23%	0.003
	B	1.45	263.2	4.14%	0.060
*Morus nigra*	A	2.85	17.6	0.28%	0.008
	B	2.08	290	4.56%	0.095
*Prunus avium*	A	1.16	15.2	0.24%	0.003
	B	3.09	373.6	5.87%	0.181
*Prunus cerasifera*	A	1.15	14.4	0.23%	0.003
	B	2.11	283.2	4.45%	0.094
*Prunus domestica*	A	1.19	12.8	0.20%	0.002
	B	2.35	207.6	3.26%	0.077
*Prunus dulcis*	A	1.23	11.2	0.18%	0.002
	B	2.35	139.6	2.19%	0.052
*Pyrus communis*	A	1.32	10.8	0.17%	0.002
	B	1.86	166	2.61%	0.048
*Sorbus domestica*	A	1.69	14.8	0.23%	0.004
	B	1.63	218	3.43%	0.056

*A* young individuals (5 year old), *B* adult individuals (30 year old)

### Ecosystem services of the “urban forest” evaluated by species

From the results obtained from the study of ecosystem functions of some fruit trees in the city, it was possible to find that over time, these species can absorb a significant amount of CO_2_ and PM10 particulate matter and lower the temperature up to 1.16 °C with the use of adult individuals. The expansion of their crown provides, especially from May through September, enough shade coverage to allow for cooling of the air around them and to lower the temperature by a few degrees Celsius under the crown relative to the surrounding area. Using the platform “Lifeclivuttreedb,” it was possible to estimate the CO_2_ stock of the tree based on DBH, height, and age over 50 years. Among the species of particular interest, we find *Juglans regia* which manages to absorb 2.33 tons 73 of CO_2_, placing itself well above the average compared to the other species taken into consideration. One of the factors that determine PM10 absorption is the structure of the leaf, its conformation, and the presence of trichomes. *Juglans regia* leaves are particularly efficient in the absorption of atmospheric particulate matter thanks to large leaf laminae with secondary veins equipped with tufts of hair on the lower page. *Juglans regia* has a total crown area larger than other individuals due to the area it can shade (107.4 m^2^/individual), and within the urban forest, it can reduce the under-crown air temperature of 1.39 °C. The roughness, shape, and size of the leaf can further influence the volumes of evaporated water and the transfer of energy. In the case of *Juglans regia*, it can absorb 4.135 Watt of energy and therefore cool the surrounding environment through evapotranspiration. Paying attention to some aspects, such as the exposition of the edible fruit woody plant, the characteristics of the soil, this species, thanks to its resistance to cold and its rusticity, could reach big dimensions and could be useful if placed in urban areas. The CO_2_ stock of *Morus nigra*, estimated by DBH and age, was 2.40 tons, the highest of all species considered in the study. *Morus nigra* also performs very well in the absorption of PM10 thanks to the characteristics of the leaves: rough and hairy on the upper leaf, which allow it to absorb 143.1 g of PM10/year for each tree. Its thick, expansive crown can shade an area of 72.5 m^2^ per individuals and reduce the air temperature under the crown by 2.08 °C. Also, *Sorbus domestica* can be considered a species with good potential in urban contexts. In fact, with a stock of 1.51 tons of CO_2_ over 50 years, an absorption of 132.7 g of PM10 per tree, and a shading of 54.5 m^2^/individual, it is one of those species that are above average. *Sorbus domestica* trees can reduce the under-crown air temperature by 1.63 °C. The species that instead expressed the most limited performances if planted in urban areas, especially considering their ecosystem values, are *Prunus avium*, *Malus sylvestris*, and *Arbutus unedo*. *Prunus avium* presents low values for PM10 absorption and for cooling by evapotranspiration since its leaf surface does not reach a great development. Because of the limited shape of the leaf laminae, it is also estimated that it can reach about 100 m^2^ at the age of 50 years, while the other species can reach a wider leaf area, even more than double. For *Malus domestica*, the only parameter above the average concerns the absorption of PM10 since, thanks to the characteristics of the globular and densely leafy crown and of the leaf (with trichomes and waxes); it is estimated that it can absorb up to 121.4 g of PM10 per tree. Finally, the species of *Arbutus unedo* is the one that has the lowest score in all the functions considered and therefore has less potential in urban areas. The *Arbutus unedo* species appears as a small tree with dense and irregular foliage and therefore absorbs little CO_2_ and PM10 compared to all the others.

## Discussion

In this work, we tried to obtain more information and data about the utilization of 16 species of fruit trees introduced in a “virtual” way, through a digital platform, in an urban context. The results described showed that the absorption capacities of CO_2_ and PM10 and the cooling potential of the environment depend mainly on the morphological characteristics of edible fruit woody plants, their height, DBH, the expansion of their crown, and the characteristics of the leaf system. The data show that some of these tree species can offer different performances depending on the environmental conditions of the cities and the pollutants present in the atmosphere. Other work has also evaluated the performance of fruit trees in urban settings evaluating CO_2_ stock. In this case, the trees of the study area sequestered a total of 38 t CO_2_ for 50 years, with 95 kg CO_2_ sequestered per resident and 2.4 kg CO_2_/ m^2^ of floor space during the whole 50 years (Ariluoma et al., 2021) showing similar data respect our estimates. Our study evidenced how even the use of fruit trees can be useful in bringing ecosystem benefits to cities and at the same time produce food that is freely available to the city community, which can collaborate in taking care of green areas if they wish, with the help of experts in the field. In fact, an “Edible Urban Forest” appropriately inserted in a particular territory will represent a green area that will also need, in some cases, maintenance activities, such as pruning or fruit picking, and that will require close collaboration between citizens, agencies, and associations, so as to reduce the costs that might arise for the presence of fruit trees. In Seattle, for example, the Seattle Department of Parks and Recreation has implemented an urban forest management plan, particularly for fruit picking. There is a program, a nonprofit organization, that strives to encourage community building by giving people a chance to garden together and learn from each other. One of the key values the program promotes is access to healthy, organic food. A principal program’s activity is to maintain plots to donate to local food banks, and the remaining fruit instead is harvested and delivered to food providers serving the city’s needy residents. There are a number of volunteers who are responsible for picking and delivering the fruit and storing the fruit-picking equipment. Started in 2005 to serve just one neighborhood, in 2009, volunteers collected and distributed more than 19,600 pounds of apples, pears, and plums throughout the city (McLain et al., 2012).

Fruit trees with a lower potential and minor fruit trees, for example, *Arbutus unedo* or *Sorbus domestica*, chosen in our work, can also contribute to increasing biodiversity, enhancing parts of the city territory. Urban Forest is based on the imitation of the forest ecosystem, respects the times of growth, and represents an authentic natural system to improve the “urban ecosystem.” The preservation and dissemination of environmental and ethnobotanical knowledge, i.e., the memory of once-used fruits passed down through the generations, can help design gardens for food use.

Several studies, such as in Nowak’s investigations (Nowak et al. 2013) and Velasco analyses (Velasco et al. 2016), found that carbon sequestration by urban vegetation is very small compared to the anthropogenic emissions determined by city areas, which was the opposite in small rural municipalities where carbon sequestration exceeded the total carbon production (Paloheimo & Salmi, 2013). To obtain important results in climate change mitigation, it is necessary for carbon to remain stored in wood for a very long time, but the same soil also has a high capacity to store carbon (Ariluoma et al., 2021). Therefore, by adding biochar in the growing medium, it will be possible to increase the carbon storage of urban soil (Ghosh et al., 2012; Scharenbroch et al., 2013), considering that biochar is able to retain more water, absorb nutrients, and influence mycorrhizal growth bringing benefits to edible woody fruit plants (Atkinson et al., 2010). With regard to what was stated above, the Urban Forest designing and realization require the collaboration of many subjects and an interest in advancing an important cause, mitigating climate change in cities by making them as resilient as possible.

## Conclusion

The present study was based on a virtual model, so it includes several uncertainties since it is not possible to predict exactly how the chosen species would behave in an urban setting, as they may be subject to various external agents. All data are purely theoretical but can serve as a starting point for real models of urban greenery management that includes the use of fruit woody species. Future investigations of the main tree species will specifically calculate their potential production yield and the economic value derived from the sale of the fruits obtained, improving the edible landscape designing and creating within cities from an environmental point of view and at the same time bringing several benefits to the entire city community.
